# Positive Outcomes Following Cervical Acceleration-Deceleration (CAD) Injury Using Chiropractic BioPhysics^®^ Methods: A Pre-Auto Injury and Post-Auto Injury Case Series

**DOI:** 10.3390/jcm12196414

**Published:** 2023-10-09

**Authors:** Tim C. Norton, Paul A. Oakley, Jason W. Haas, Deed E. Harrison

**Affiliations:** 1 Private Practice, Shoreline, WA 98133, USA; 2 CBP Nonprofit, Eagle, ID 83616, USA; drjasonhaas@gmail.com (J.W.H.); drdeedharrison@gmail.com (D.E.H.); 3 Kinesiology and Health Science, York University, Toronto, ON M3J 1P3, Canada; 4 Private Practice, Newmarket, ON L3Y 8Y8, Canada

**Keywords:** motor vehicle collision, whiplash, cervical lordosis, subluxation, case series, chiropractic

## Abstract

This series illustrates how rear-end impact motor vehicle collisions (MVCs) alter the cervical spine’s alignment and demonstrates therapeutic use of cervical extension traction to improve lordotic alignment and other outcomes. This is a retrospective reporting of 7 adult patients (4 males and 3 females, 28–42 years) treated for cervical hypolordosis. These subjects received Chiropractic BioPhysics^®^ (CBP^®^) rehabilitation and then were involved in a rear-end MVC. All cases had radiographic assessment that quantified the buckling of the cervical spine, presumably resulting directly from the CAD trauma. After an average of 3 years and 9 months (range: 1–7.6 years) following their initial program of care, the 7 patients sought care for a second time after the MVC. At this time, compared with their previously recorded post-treatment spine radiographs, there was an average 18.7° (range: 7.6–35.4°) reduction in cervical lordosis, a 9.2 mm (range: 3.6–19.8 mm) increase in anterior head translation (AHT), an 11.3° (range: 0.2–19.9°) decrease in the atlas plane line (APL), as well as a 35.7% (range: 22–52%) average neck disability index score (NDI) measured after the MVC. After the crash, a second round of CBP rehabilitation was administered, resulting in an average 15.1° improvement in cervical lordosis, 10.9 mm reduction in AHT, 10.4° increase in APL, and a 23.7% drop in NDI after an average of 35 treatments over 9 weeks. Treatment was universally successful, as an average 80% re-establishment of the lordosis toward its pre-injury state was found. There were no adverse events reported. This case series demonstrates that motor vehicle collisions may alter the alignment of the cervical spine. Rehabilitation of the cervical curve using extension traction improved the patients’ initial pre-crash alignments toward their pre-injury alignments and was likely responsible for improvement in the patients’ conditions. Clinical trials are needed to confirm these findings.

## 1. Introduction

Cervical acceleration-deceleration (CAD) injury of the spine is common after rear-end collisions [1]. It has been shown that rear-end impacts cause an instant cervical spine alteration in alignment involving a posterior translation of the head followed by a hyper-extension and then hyper-flexion of the neck [2,3]. Indeed, intersegmental motions can be complex and contribute to individual-level specific injury, spine misalignment, and abnormal functioning [4,5,6,7,8,9]. This abnormal spine alignment may persist for years afterward, resulting in pain, decreased patient reported outcomes (PROs), and increased likelihood of future disability [10,11,12,13].

Traditional CAD treatment guidelines do not specifically consider treating the alignment of the cervical spine. However, recent case reports have documented successful outcomes in re-establishing the cervical lordosis in patients involved with previous whiplash-associated disorders (WADs) (e.g., Fedorchuk et al. [14]). Although traditionally it has been debated whether the cervical spine alignment is important in the etiology of WADs [15,16], recent evidence points to the cervical spine alignment being an important predictor of patient recovery [17].

In 2006, Harrison et al. [18] reviewed the results of 41 consecutive patient cases following MVCs. Here, initial patient records and cervical spine lateral radiographs within 30 days prior to the crash were obtained in order to identify possible cervical lordosis alterations resulting from the crash. It was found that the crash created an average 10° loss of the cervical curve and an altered geometric curve in the form of segmental extension in the lower, flexion in the middle, and extension in the upper cervical spine. This is termed a second-order buckling configuration in the snap-through buckling of columns. In a population of WAD injured persons, Rydman et al. [17] were the first to consider the alignment metrics of the neck tilt and thoracic inlet angle (TIA), values that are constitutional and therefore patient-specific [19]. In assessing 46 patients’ self-reported recoveries after WADs, of a 28% nonrecovery rate, 50% had low neck tilt versus 8% having high neck tilt, and 50% had a low TIA versus 14% having a high TIA [17]. Thus, Rydman et al. showed the definitive existence of an association between recovery after a WAD and the constitutional sagittal alignment of the cervical spine [17].

Although there are case reports showing the rehabilitation of traumatic snap-through buckling (e.g., straightened cervical spine) in patients who have had a CAD injury [14], importantly, no case has quantified the sagittal cervical spine alignment pre- and post-collision and then shown a re-establishment of the lordosis post-rehabilitation. Whereas the Harrison et al. [18] case series ascertained that the loss of cervical lordosis at patient presentation was indeed partially resulting from the CAD event, no treatment was provided, so it remains unclear whether or not this crash-induced altered geometric curve alignment is amendable to spine rehabilitation. There is a need, therefore, for a real-life capture of the known cervical alignment prior to and then immediately following an MVC and its demonstrated correction using contemporary lordosis altering methods.

The purpose of this case series is to illustrate how a rear-impact CAD injury alters the cervical spine. Furthermore, we demonstrate how cervical extension traction (CET) as a part of Chiropractic BioPhysics^®^ (CBP^®^) structural rehabilitation methods can re-establish the lordotic alignment and facilitate patient recovery.

## 2. Materials and Methods

This is a retrospective reporting of 7 adult patients who had been previously treated for cervical spine altered alignment (straightening of the cervical spine) and who had increased their lordosis after receiving CBP rehabilitation and then were involved in a whiplash event. All cases had radiographic assessment that quantified the buckling of the cervical spine, presumably resulting directly from the CAD whiplash event. All patients then received CBP rehabilitation procedures for a second time and again demonstrated a re-establishment of the cervical lordosis.

CBP is a full spine and posture corrective rehabilitation program that involves mirror image^®^ (MI) exercises, adjustments, and spinal traction (www.idealspine.com (accessed 9 September 2023)). Mirror image refers to stressing the spine and posture into the reverse of the presenting misalignment. Spinal traction for the cervical spine involves cervical hyperextension traction to increase the lordosis, and there are many clinical trials showing the efficacy of cervical spine extension traction at increasing the cervical lordosis [20]. The main types of cervical extension traction include the original “compression-extension”, a seated “Pope’s 2-way”, a seated “2-way compression-extension”, as well as a cervical extension traction orthotic, the Denneroll orthotic (Denneroll Spinal Orthotics, Wheeler Heights, NSW, Australia) [20].

In addition to the numerical pain rating scale (NPRS) pain questionnaire [21,22] and the neck disability index (NDI) [23,24], all patients were diagnosed with cervical spinal snap-through buckling (i.e., loss of the normal lordosis) by quantifying the cervical alignment on lateral (sagittal view) cervical X-rays taken in a standing neutral position. For reference, a normal C2–7 absolute rotation angle (ARA) cervical lordosis (as measured by the posterior tangent angle [25,26]) should ideally be in the range of 29–40° [27] or at least be greater than 25° [27]. The PostureRay^®^ EMR system (PostureCo., Trinity, FL, USA) was used to digitize the images. The absolute rotation angle (ARA) between the posterior margins of the vertebral body of C2 and C7 was used to measure the global lordosis. The anterior head translation (AHT) was quantified by the horizontal distance between the vertical line projected from the posterior inferior corner of C7 and the offset of the posterior superior aspect of C2’s body (excluding the dens). The atlas plane line (APL) was measured as the angle between the horizontal line and the best fit line of approximating the midline of C1. These mensuration methods are reliable and repeatable with a standard error of measure of less than 2° [26].

All patients received full spine manipulation (i.e., high-velocity, low-amplitude spinal manipulative therapy (SMT)) as well as CBP mirror image posture adjusting for forward head posture (FHP). The details of the patient demographic and radiographic metrics are summarized in Table 1 and Table 2, respectively. All patients gave verbal and written consent prior to the reporting of their clinical outcomes. The clinical details of individual patient presentations and treatment specifics are discussed below.

### 2.1. Case 1

**Figure 1:**
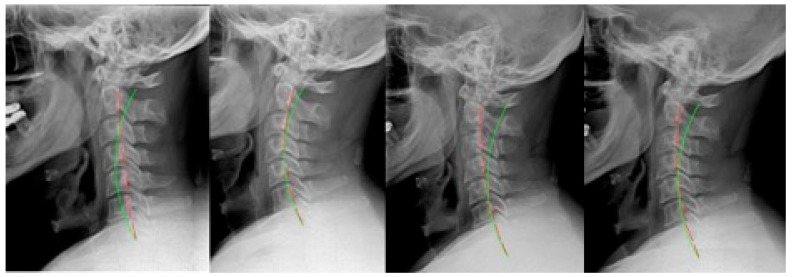
Case 1: (**left**) pre-treatment; (**middle left**) post-treatment; (**middle right**) post-MVC (pre-second treatment); and (**right**) post-MVC treatment. Red line is patient; green line is normal.

Case 1 (Figure 1) was a 42 year-old male originally seen following an MVC. The details of the collision include the patient being the driver with a seat belt on when they were stopped and being hit from behind by another vehicle moving at a high rate of speed. The patient was not aware of the impact. The original treatment plan included 35 treatments over 9 weeks at a frequency of 4 times per week. From the 9th treatment onward, cervical extension traction involving seated compression traction was performed, and Denneroll extension traction was prescribed for home use 3 weeks into care. The patient discontinued care after treatment.

The patient was not seen until 7 years and 7 months later, when they presented following an MVC where they were the driver while wearing a seatbelt and were hit from behind into another vehicle in front. They were aware of the impact. The second round of treatment consisted of 23 visits over 6 weeks. There were 23 sessions using seated compression traction, and home Denneroll traction was prescribed after the first week to be performed daily at home.

### 2.2. Case 2

**Figure 2:**
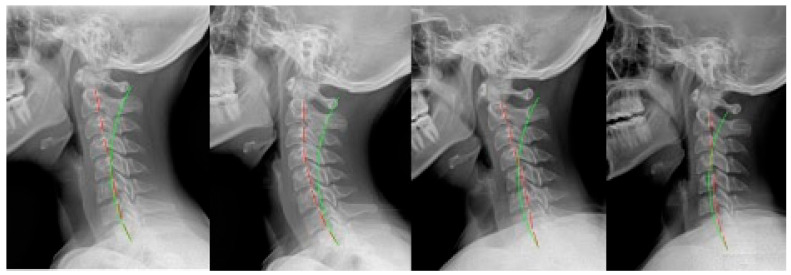
Case 2: (**left**) pre-treatment; (**middle left**) post-treatment; (**middle right**) post-MVC (pre-second treatment); and (**right**) post-MVC treatment. Red line is patient; green line is normal.

Case 2 (Figure 2) was a 28 year-old female first presenting for care for a non-MVC-related complaint. The first round of CBP rehabilitation consisted of 35 visits over 9 weeks. There were 29 sessions of seated compression traction, and daily home Denneroll traction was prescribed after 2 weeks. Following this program, the patient remained under maintenance care at a frequency of once per month for 9 months.

After 5 years and 5 months, this patient re-presented for care following an MVC. She was a passenger while wearing her seatbelt, and the car was hit from behind while in a stopped position. The patient was aware of the impact. The new round of treatments numbered 51 over 13 weeks. For the 6th session, the patient received Pope 2-way extension traction for 30 sessions and then was transitioned to compression traction for the remaining 16 sessions.

The patient was prescribed head retraction exercises for 21 sessions, and daily home Denneroll traction was prescribed after 3 weeks.

### 2.3. Case 3

**Figure 3:**
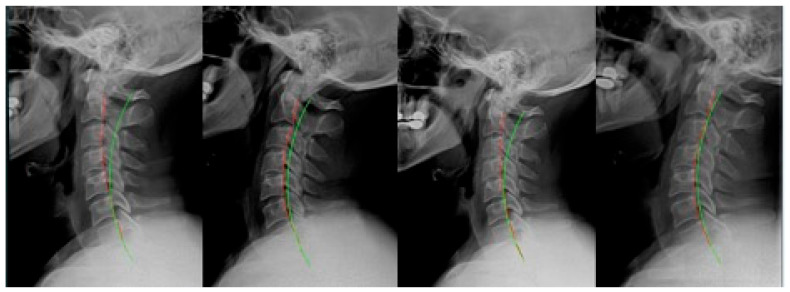
Case 3: (**left**) pre-treatment; (**middle left**) post-treatment; (**middle right**) post-MVC (pre-second treatment); and (**right**) post-MVC treatment. Red line is patient; green line is normal.

Case 3 (Figure 3) was a 39 year-old male presenting with non-MVC complaints. He received 23 sessions over 6 weeks. For the 6th visit, the patient received seated compression traction, which was applied for the remainder of the treatments. Home daily Denneroll traction was prescribed at the 2 week mark. After treatment, the patient remained on a once-per-month maintenance program receiving adjustments only.

One year later, the patient presented with new complaints following an MVC. The patient was driving with a seatbelt on and was struck while slowing down by a vehicle that was speeding up from behind. The patient was aware of the impending collision. The patient received 23 sessions over 6 weeks. On the third visit, they received seated compression traction which continued thereafter. After the first week, daily home Denneroll traction was prescribed. Ten sessions of head retraction exercises were also performed.

### 2.4. Case 4

**Figure 4:**
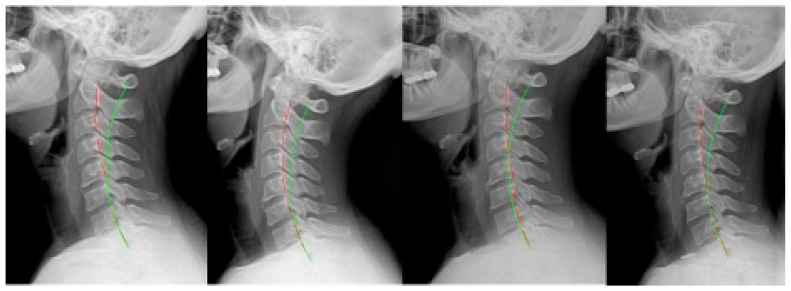
Case 4: (**left**) pre-treatment; (**middle left**) post-treatment; (**middle right**) post-MVC (pre-second treatment); and (**right**) post-MVC treatment. Red line is patient; green line is normal.

Case 4 (Figure 4) was a 34 year-old female who first presented for treatment of symptoms related to an MVC. She originally received 35 treatments over 9 weeks. After 10 sessions, the patient received 25 sessions involving seated compression traction. After 4 weeks, daily home Denneroll traction was prescribed. After treatment, the patient remained on a once-per-month maintenance program of adjustments only.

Three years and 4 months later, the patient presented following an MVC. They were a passenger while wearing a seatbelt, and the vehicle was struck from behind while in a stopped position. The patient was unaware of the impending collision. The patient received 23 treatments over 6 weeks. The last 19 sessions involved seated compression traction. Daily home Denneroll traction was prescribed after the first week.

### 2.5. Case 5

**Figure 5:**
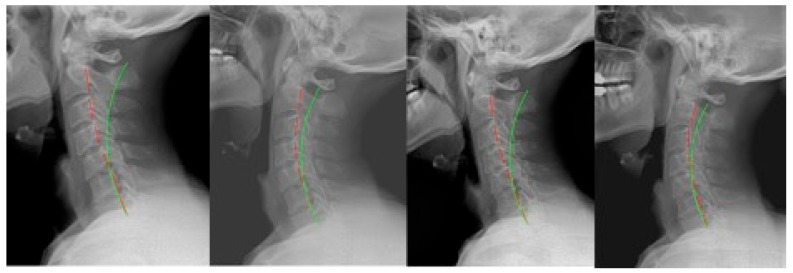
Case 5: (**left**) pre-treatment; (**middle left**) post-treatment; (**middle right**) post-MVC (pre-second treatment); and (**right**) post-MVC treatment. Red line is patient; green line is normal.

Case 5 (Figure 5) was a 52 year-old male originally presenting with non-MVC complaints. He was originally treated for 35 sessions over 9 weeks. From the 4th visit onward, he was treated with Pope 2-way traction, and home Denneroll traction was prescribed to be performed daily after the 3rd week. The patient remained on a monthly treatment program, receiving adjustments only after completing the rehabilitation program.

Three years and 6 months later, the patient presented following an MVC. As a passenger, he was wearing a seatbelt and was struck from behind while stopped. He was not aware of the impending collision. He was treated 35 times over 9 weeks. From the fifth session onward, the patient performed Pope 2-way traction. Home Denneroll traction was prescribed after the first week.

### 2.6. Case 6

**Figure 6:**
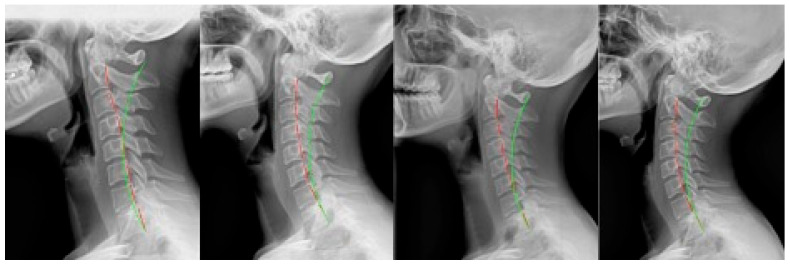
Case 6: (**left**) pre-treatment; (**middle left**) post-treatment; (**middle right**) post-MVC (pre-second treatment); and (**right**) post-MVC treatment. Red line is patient; green line is normal.

Case 6 (Figure 6) was a 32 year-old female who originally presented with a non-MVC complaint. The patient was treated 35 times over 9 weeks and performed 30 sessions of seated compression traction. Home Denneroll traction was prescribed after the 3rd week. The patient was treated once per month for adjustments only after rehabilitation.

After 2 years and 1 month, the patient reported after an MVC as they were struck from behind while stopped. She was wearing her seatbelt but was not aware of the impending collision. The patient then received 36 treatments over 9 weeks, including 31 sessions of seated compression traction. Daily home Denneroll traction was prescribed after the second week.

### 2.7. Case 7

**Figure 7:**
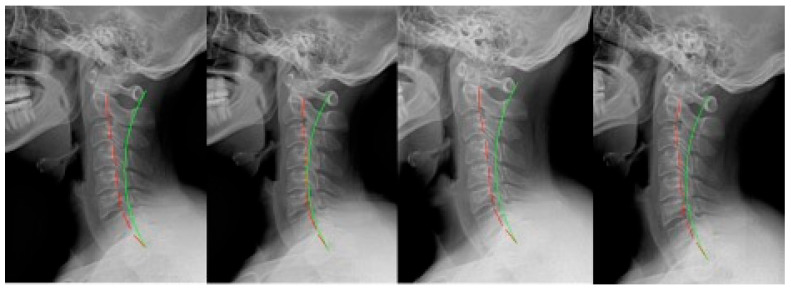
Case 7: (**left**) pre-treatment; (**middle left**) post-treatment; (**middle right**) post-MVC (pre-second treatment); and (**right**) post-MVC treatment. Red line is patient; green line is normal.

Case 7 (Figure 7) was a 36 year-old male originally presenting following an MVC. He was the driver in a head-on collision. He was wearing a seatbelt, moving at the time of impact, and was aware of the impending collision. The patient received 47 treatments over 12 weeks, including 37 sessions of seated compression traction. There were also 9 sessions involving head retraction exercises. Daily home Denneroll traction was prescribed after 4 weeks of care. The patient continued treatment on a maintenance basis of 2 times per month for 6 months and then once per month thereafter, receiving adjustments only.

Three years and 3 months after the initial care, the patient presented following a second MVC. The patient was the driver while wearing a seatbelt and was struck from behind while stopped. They were aware of the impending collision. The patient received 51 treatments over 13 weeks, and this included 47 sessions of compression traction. There were also 21 sessions of head retraction exercises, and daily home Denneroll traction was prescribed after the first 3 weeks.

## 3. Results

Of the seven patients included in this series, four were male, and three were female. The average age was 37.6 ± 7.8 years (range: 28–52 years), the average height was 174.5 ± 12.8 cm, the average weight was 81.3 ± 11.7 kg, and the average BMI was 26.8 ± 4.1 kg/m^2^ (Table 1). At the initial examination, three originally presented for treatment of symptoms following an MVC, and four presented with non-MVC-related complaints. Of these patients, 6/7 had lower back pain, 4/7 had neck pains, and 3/7 had headaches (Table 1). Following the MVC, and prior to the second round of treatment, all patients suffered from MVC-related neck pains, with three also having LBP and five also having headaches (Table 1).

The first round of treatments showed that the patients provided a program of CBP rehabilitation demonstrated an average 20.1° (range: 6.1–25.8°) improvement in cervical lordosis, a 9.4 mm (range: 3.3–19 mm) reduction in AHT (TzH), an 11.7° (range: 3.7–12.9°) increase in APL, an 18% (range: 0–46%) drop in NDI, and a 3.3 (range: 0–6/10) point drop in pain intensity following an average of 35 treatments over 9 weeks (Table 2). Notably, the average pre-treatment lordosis was −13.2°, which is in the pathological range, while the post-treatment lordosis averaged 33.3°, which is within the normative range [27]. Following this rehabilitation program, six of the seven patients remained on a maintenance program of monthly treatments, receiving spinal manipulation only.

After an average of 3 years and 9 months following their initial program of care (range: 1–7.6 years), the 7 patients sought care for a second time due to being in an MVC. At this time, compared with their post-treatment spine radiographs, there was an average 18.7° reduction in cervical lordosis (range: 7.6–35.4°), a 9.2 mm (range: 3.6–19.8 mm) increase in AHT (TzH), and an 11.3° (range: 0.2–19.9°) decrease in APL (Table 2). There was also a 30.6% (range: 20–40%) average increase in neck disability as well as a 5.3 (range: 3.5–8/10) point average increase in pain intensity. These values indicate that the vehicle collision altered the sagittal cervical spine parameters for the worse and contributed to neck pain and disability (Table 3).

Following the MVC, all subjects underwent a second round of CBP rehabilitation. Herein, there was an average 15.1° (range: 7.8–23.1°) improvement in cervical lordosis, a 10.9 mm (range: −2.7–24.6 mm) reduction in AHT (TzH), a 10.4° (range: 4.3–14.5°) increase in APL, a 23.7% (range: 12–40%) drop in NDI, and a 5.3 (range: 3–8/10) point drop in pain intensity after an average of 35 treatments over 9 weeks (Table 4). Also, the 5/7 patients having headaches experienced relief. There were no adverse events reported throughout treatment. Notably, the average lordosis after the MVC but prior to the second round of treatments averaged −14.6°, which is within the pathological range, and after treatment, it averaged −29.7°, which is within the normative lordosis range [27]. Compared with the pre-MVC lordosis values, the cervical rehabilitation resulted in an average of 80% correction (15.5° average correction post-MVC/18.7° average loss of lordosis post-MVC = 81%) toward pre-injury status; in other words, in the 7 patients, the treatment resulted in an average of 20% less lordosis than the definitively known pre-MVC values.

## 4. Discussion

This report has documented that MVCs resulted in an average 18.7° loss in cervical lordosis, a 9.2 mm increase in AHT, and an 11.3° reduction in APL in these 7 patients. Lordosis-promoting rehabilitation was successful at achieving an average 15° improvement in lordosis and an overall average curve of 30°, which is within the normative range for lordosis as measured by the Harrison posterior tangential method [27]. There was a coinciding 10.9 mm reduction in AHT, a 10.4° increase in APL, and importantly, a 24% reduction in disability and 5.3/10 point reduction in pain intensity. This case shows that structural rehabilitation to the cervical spine is possible following an MVC and represents the first series showing successful cervical structural rehabilitation both prior to and following an MVC event.

The clinical outcomes of the series of seven patients reported herein demonstrate the biomechanical impact of a rear-impact CAD event on the cervical spine. Rear-end motor vehicle impacts may instantly buckle the cervical spine, leading to the loss of a normal lordotic alignment. This series also demonstrates the consistency and reproducibility of CBP methods, which includes the unique application of cervical extension traction to reestablish the cervical lordosis. Since symptoms were associated with loss of the cervical lordosis in some patients pre-whiplash, as well as in all patients post-whiplash, this series also adds evidence that points to loss of the cervical spine lordosis as a generator of cranio-cervical symptoms, including that associated with the loss of lordosis following whiplash.

Often, a spine practitioner evaluates a patient following an MVC, and the diagnosis of cervical spine subluxation (e.g., hypolordosis, straight, and kyphosis) may be made. However, a comparison to previous X-rays to verify MVC-induced subluxation is often not possible. Therefore, it is often assumed that an MVC causes a “snap-through” buckling (i.e., conforms to a non-lordotic position) of the cervical spine during the injury event without definitive proof. This series adds evidence to the biomechanically understood classic CAD event that alters the alignment of the cervical spine, both globally and intersegmentally [2,3,4]. This series of patients is also consistent with the only previous investigation that could be identified on the topic of alterations of the cervical lordotic curve in patients with an initial pre-injury X-ray and a post-MVC lateral cervical x-ray within 1 month of the crash. Following an MVC, Harrison et al. [18] identified a 10° average reduction in the cervical curve (ARA C2–C7), a decreased APL, a slight increase in AHT, and an alteration in the geometric alignment of the cervical curve consistent with a second-order buckling alignment. Herein, our results of the seven patients were similar to those reported by Harrison et al. [18].

Long-term sequelae following CAD injury have been documented and include pain, disability, and poor quality of life [1]. Since cervical spine subluxation may occur after a CAD injury, and that altered cervical alignment has been shown to be associated with many typical symptoms related to whiplash injury, including neck pain, headache, and radiculopathy, among other symptoms [28,29,30,31,32,33,34,35], it seems imperative to screen the cervical spine for its radiographic sagittal alignment. Importantly, it should be known that the sagittal cervical spine alignment is not always adequately assessed by medical radiologists (i.e., not measured), and caution should be taken in radiological interpretation. Images should be directly measured using reliable methods (e.g., C2–C7 ARA or C2–C7 Cobb).

It should be noted that all patients received multimodal care including cervical extension traction, SMT, and drop table mirror image head retraction adjustments, and some patients performed head retraction exercises. Although it may seem to complicate the process of distinguishing the specific treatment leading to an improved lordosis, multiple trials have detailed that extension traction specifically results in an improved lordosis, whereas other physiotherapeutic treatments do not [20]. Also, this series reports actual clinical data. Therefore, it was impossible to limit treatment to individual patients in the real clinical encounter.

Extension traction methods are thought to create a hyperextension of the neck and elongate the anterior portion of the spine’s soft tissues, including the longitudinal ligament and anterior portion of the intervertebral discs and anterior muscles. This traction effect causes a deformation in the soft tissues (i.e., muscles, ligaments, and discs), and when subjected to continuous loads, various biomolecular processes including creep relaxation and viscoelastic deformation occur [36,37,38]. Biomechanical elongation of the anterior structures may lead to permanent structural tissue resting length changes, and when performed in a frequent manner (i.e., daily or three times per week), this results in sagittal spine alignment improvements over time [20].

Alterations to the sagittal cervical spine alignment has been shown to predict poor outcomes after MVCs. Norris and Watt [10] determined two features important in predicting poor outcomes after acute neck injury resulting from MVCs: (1) the presence of early degenerative spondylosis and (2) loss of the normal lordosis in the cervical spine. Hohl [11] determined that reversal of the cervical spine (i.e., kyphosis) was associated with the development of degenerative intersegmental disc disease within 5 years after an MVC. It is known that altered loading of the cervical spine, such as that caused by non-lordotic positions, will cause abnormal stresses and strains and lead to the development of degenerative changes [39,40]. Thus, it seems that cervical extension traction methods as a part of the CBP technique may offer a unique and specific modality in the treatment of MVC-related alterations to the cervical curvature.

This case has demonstrated that in the select cases presented here, CBP methods including extension traction restored the cervical lordosis and reduced symptoms in MVC-injured patients. Cervical extension traction, as used in a multimodal cervical spinal rehabilitation protocol, has established repeatability when used in the treatment of multiple cervical spine disorders, including cervical myofascial pain syndrome, cervicogenic dizziness, cervical radiculopathies, and neck pains [20]. Indeed, relief of symptoms was shown to occur after an average of 12–18° lordosis correction over 5–15 weeks [20]. Interestingly, the amount of structural change in these various clinical trials was close to what has been reported in dozens of cervical case reports. Finally, since it has been shown that whiplash patients demonstrate increased forward head posture and worse sensorimotor control [41], and since these treatment methods can improve structural alignment and neurological measures, the similarity of CBP case reporting to the repeatability of the clinical trial data supports these methods as a potentially useful treatment for cervical hypolordosis or kyphosis after whiplash. It is our hope to create awareness that a validated, reliable and preexisting treatment protocol could prove to be an effective treatment for this unique population.

### 4.1. Limitations

There are limitations to this case series. The main limitations are the small sample size (*n* = 7) and the retrospective nature of the report. Also, all assessments and treatments were provided by the same doctor (the first author). The time duration between the sagittal cervical X-ray that preceded and the one taken following the MVC event, in most cases, was multiple years (range: 1–7.6 years). This opens up the possibility that other non-MVC events may have contributed to the loss in the cervical curve. It should be known, however, that the cervical alignment is relatively stable over time. In fact, many studies have shown the cervical spine and head positioning to remain in stable alignment for years, unless an injury occurs or treatment is pursued [42,43].

### 4.2. Conclusions

This case series demonstrates that motor vehicle collisions directly alter the alignment of the cervical spine. Rehabilitation of the cervical curve using extension traction restored the patients’ post-crash altered alignments back toward the pre-injury alignments and was likely responsible for improvement in the patients’ conditions. Biomechanical models and clinical trials are needed to confirm the findings reported herein.

## Figures and Tables

**Table 1:** Patient demographic and symptom data.

	Case 1	Case 2	Case 3	Case 4	Case 5	Case 6	Case 7
Age (year)	42	28	39	34	52	32	36
Sex	M	F	M	F	M	F	M
Height (cm)	185.4	154.9	182.9	170.2	190.5	162.6	175.3
Weight (kg)	74.8	81.6	99.8	74.8	88.5	63.5	86.2
BMI (kg/m^2^)	21.8	34	29.8	25.8	24.4	24	28.1
First txt complaints	LBP	LBP, HA	NKP, LBP	NKP, LBP, HA	LBP	NKP	NKP, LBP, HA
Post-collision complaints	NKP, HA	NKP, HA	NKP, LBP	NKP, HA	NKP, LBP, HA	NKP, HA	NKP, LBP

Note: LBP = low back pain; NKP = neck pain, HA = headaches.

**Table 2:** Pre- and post-cervical radiographic parameters and neck disability from first trial of rehabilitation (prior to later motor vehicle collision).

		Case 1	Case 2	Case 3	Case 4	Case 5	Case 6	Case 7	Avg (SD)
Pre- rehab	ARA	−12.5°	−0.7°	−11.7°	−23.0°	−14.8°	−5.9°	−23.7°	−13.2° (8.4°)
	TzH	13.6 mm	21.6 mm	17.2 mm	18.6 mm	35.6 mm	23.3 mm	20.6 mm	21.5 mm (7.0 mm)
	APL	−13.9°	−14.4°	−25.6°	−17.7°	−17.6°	−14.1°	−21.4°	−17.8° (4.4°)
	NDI	0%	0%	18%	52%	0%	30%	62%	23.1% (25.9%)
	Pain	0/10	0/10	5/10	5/10	4/10	6/10	7/10	3.9/10 (2.8)
	HA pain	0/10	4/10	0/10	7/10	0/10	0/10	3–4/10	* 4.8/10 (1.9)
Txt details	No./time	35/9 w	35/9 w	23/6 w	35/9 w	35/9 w	35/9 w	47/12 w	35/9 w
Post- rehab	ARA	−37.6°	−26.3°	−36.0°	−36.6°	−35.4°	−31.7°	−29.8°	−33.3° (4.2°)
	TzH	8.1 mm	18.3 mm	1.9 mm	9.2 mm	16.6 mm	17.2 mm	12 mm	11.9 mm (6.0 mm)
	APL	−23.9°	−27.3°	−34.0°	−28.3°	−28.4°	−25.7°	−25.1°	−27.5° (3.3°)
	NDI	0%	0%	2%	8%	0%	10%	16%	5.1% (6.3%)
	Pain	0/10	0/10	0/10	2/10	1/10	0/10	1.5/10	0.6/10 (0.9)
	HA pain	0/10	0/10	0/10	0/10	0/10	0/10	0/10	0/10 (0.0)

Note: ARA = C2–7 absolute rotation angle, TzH = forward head posture, APL = atlas plane line, NDI = neck disability index. * Average of those with headaches (*n* = 3).

**Table 3:** Pre- and post-motor vehicle collision cervical radiographic parameters and neck disability.

		Case 1	Case 2	Case 3	Case 4	Case 5	Case 6	Case 7	Avg (SD)
Pre- MVC (Post-rehab)	ARA	−37.6°	−26.3°	−36.0°	−36.6°	−35.4°	−31.7°	−29.8°	−33.3° (4.2°)
	TzH	8.1 mm	18.3 mm	1.9 mm	9.2 mm	16.6 mm	17.2 mm	12.0 mm	11.9 mm (6.0 mm)
	APL	−23.9°	−27.3°	−34.0°	−28.3°	−28.4°	−25.7°	−25.1°	−27.5° (3.3°)
	NDI	0%	0%	2%	8%	0%	10%	16%	5.1% (6.3%)
	Pain	0/10	0/10	0/10	2/10	1/10	0/10	1–2/10	0.6/10 (0.9)
	HA pain	0/10	0/10	0/10	0/10	0/10	0/10	0/10	0/10 (0)
Time lapse	Year, month	7 years, 7 months	5 years, 5 months	1 year	3 years, 4 months	3 years, 6 months	2 years, 1 months	3 years, 3 months	3 years, 9 months
Post- MVC (Pre-2nd rehab)	ARA	−17.7°	+9.1°	−20.3°	−18.1°	−15.4°	−17.3°	−22.2°	−14.6° (10.7°)
	TzH	18.7 mm	26.6 mm	11.3 mm	12.8 mm	36.4 mm	21.2 mm	20.9 mm	21.1 mm (8.5 mm)
	APL	−10.6°	−7.4°	−23.3°	−14.8°	−12.8°	−25.5°	−18.7°	−16.2° (6.7°)
	NDI	26%	26%	22%	48%	40%	36%	52%	35.7% (11.6%)
	Pain	7/10	8/10	3.5/10	6/10	5/10	5/10	7/10	5.9/10 (1.5)
	HA pain	7.5/10	2.5/10	0/10	6/10	7.5/10	2.5/10	0/10	* 5.2/10 (2.5)

Note: ARA = C2–7 absolute rotation angle, TzH = forward head posture, APL = atlas plane line, NDI = neck disability index; HA = headache. * Average of those with headaches (*n* = 5).

**Table 4:** Pre- and post-cervical radiographic parameters and neck disability from second trial of rehabilitation after motor vehicle collision.

		Case 1	Case 2	Case 3	Case 4	Case 5	Case 6	Case 7	Avg (SD)
Post- MVC (Pre-2nd rehab)	ARA	−17.7°	+9.1°	−20.3°	−18.1°	−15.4°	−17.3°	−22.2°	−14.6° (10.7°)
	TzH	18.7 mm	26.6 mm	11.3 mm	12.8 mm	36.4 mm	21.2 mm	20.9 mm	21.1 mm (8.5 mm)
	APL	−10.6°	−7.4°	−23.3°	−14.8°	−12.8°	−25.5°	−18.7°	−16.2° (6.7°)
	NDI	26%	26%	22%	48%	40%	36%	52%	35.7% (11.6%)
	Pain	7/10	8/10	3.5/10	6/10	5/10	5/10	7/10	5.9/10 (1.5)
	HA pain	7.5/10	2.5/10	0/10	6/10	7.5/10	2.5/10	0/10	* 5.2/10 (2.5)
Txt details	No./time	23/6 w	51/13 w	23/6 w	23/6 w	35/9 w	36/9 w	51/13 w	34.6/8.9 w
Post- MVC rehab	ARA	−30.0°	−14.0°	−37.8°	−28.6°	−35.1°	−32.7°	−30.0°	−29.7° (7.7°)
	TzH	11.7 mm	10.3 mm	−3.7 mm	15.5 mm	11.8 mm	13.9 mm	11.6 mm	10.2 mm (6.3 mm)
	APL	−20.1°	−21.9°	−36.1°	−19.1°	−23.9°	−35.0°	−29.8°	−26.6° (7.1°)
	NDI	0%	8%	10%	22%	20%	12%	12%	12% (7.4%)
	Pain	1.5/10	0/10	0.5/10	1/10	0.5/10	1/10	0.5/10	0.6/10 (0.5)
	HA pain	0/10	0/10	0/10	0/10	0/10	0/10	0/10	0/10 (0.0)

Note: ARA = C2–7 absolute rotation angle; TzH = forward head posture; APL = atlas plane line; NDI = neck disability index; HA = headache. * Average of those with headaches (*n* = 5).

## Data Availability

The datasets analyzed in the current study are available from the corresponding author upon reasonable request.
